# Experimental and Theoretical Investigation of Crystallographic Orientation Dependence of Nanoscratching of Single Crystalline Copper

**DOI:** 10.1371/journal.pone.0131886

**Published:** 2015-07-06

**Authors:** Yanquan Geng, Junjie Zhang, Yongda Yan, Bowen Yu, Lin Geng, Tao Sun

**Affiliations:** 1 The State Key Laboratory of Robotics and Systems, Robotics Institute, Harbin Institute of Technology, Harbin, Heilongjiang, 150008, P. R. China; 2 Center for Precision Engineering, Harbin Institute of Technology, Harbin, Heilongjiang, 150001, P. R. China; 3 School of Materials Science and Engineering, Harbin Institute of Technology, Harbin, Heilongjiang, 150001, P. R. China; Massachusetts Institute Of Technology, UNITED STATES

## Abstract

In the present work, we perform experiments and molecular dynamics simulations to elucidate the underlying deformation mechanisms of single crystalline copper under the load-controlled multi-passes nanoscratching using a triangular pyramidal probe. The correlation of microscopic deformation behavior of the material with macroscopically-observed machining results is revealed. Moreover, the influence of crystallographic orientation on the nanoscratching of single crystalline copper is examined. Our simulation results indicate that the plastic deformation of single crystalline Cu under the nanoscratching is exclusively governed by dislocation mechanisms. However, there is no glissile dislocation structure formed due to the probe oscillation under the load-controlled mode. Both experiments and MD simulations demonstrate that the machined surface morphologies in terms of groove depth and surface pile-up exhibit strong crystallographic orientation dependence, because of different geometries of activated slip planes cutting with free surfaces and strain hardening abilities associated with different crystallographic orientations.

## Introduction

Nanostructures with unique properties not only provide novel applications in fields of optoelectronics, sensors, transducers and biomedical sciences [1–5], but also are of significant fundamental research interests at the nanometer scale. Surface quality in terms of surface morphology and subsurface damage is one important issue governing potential application and performance of nanostructures [6–8]. Thus, how to fabricate nanostructures with ultra-fine surface finish becomes critical and challenging. The atomic force microscopy (AFM) probe-based mechanical nanoscratching has been demonstrated to be one powerful fabrication technique for functional nanostructures [9–10]. To facilitate surface quality improvement of the as-fabricated nanostructures, a fundamental understanding of the underlying machining mechanisms involved in the probe-based nanoscratching process is essentially required.

Given the ultra-small material removal thickness, the properties of workpiece materials play increased even dominant role in the probe-based nanoscratching. For instance, nanocrystalline metals of unique chemical, physical and mechanical properties not only provide novel possibilities for the fabrication of advanced functional nanostructures [11], but also bring new challenges in understanding their nanoscratching mechanisms because of internal textured microstructures [12–14]. Due to comparable probe radius with average grain size, a typical scratching pass of nanocrystalline materials under the probe-based nanoscratching involves multiple grains of different crystallographic orientations. It’s known that the mechanical properties of an anisotropic material vary with orientation, which consequently results in difficulty in obtaining a uniformly-machined surface quality over individual grains. Since the deformation of nanocrystalline materials at the grain level mainly relies on the orientation of individual grains, it’s important to examine the effect of crystallographic orientation on the nanoscratching process. Previous experiments and molecular dynamics (MD) simulations of tension [15–16], compression [17], bending [18–20] and indentation [21–22] tests under the uniaxial stress state have well documented that the crystallographic orientation-dependent mechanical response of single crystalline face centered cubic (FCC) metals is determined by the activation of {111}<110> slip systems. However, the localized multi-axis stress state in nanoscratching causes more complex tribological response with respect to the crystallographic orientation than the uniaxial stress state, which thus requires new insights above previous documentation of the crystallography dependence. On the experimental side, it is found that there is rather limited information known about the influence of crystallographic orientation on the nanoscratching. Furthermore, the influence of scratching pass number on the crystallographic orientation dependence is largely unknown. On the theoretical side, although the effect of crystallographic orientation on the nanoscratching has been investigated by several MD simulations [23–25], most of which exclusively mimicked the nanoscratching under the displacement-controlled mode, i.e., probe penetrating into sample surface with constant velocities. However, the probe-based nanoscratching experiments are usually carried out in the load-controlled mode by applying constant normal load on the probe. Furthermore, most of the probes considered in previous MD simulations are conical- or spherical-shaped, which are significantly different from the triangular pyramidal geometry that is widely used in nanoscratching experiments. Those discrepancies of nanoscratching methodologies inevitably introduce uncertainties in the comparison between experimental data and simulation results.

Therefore, in the present work we conduct experiments and MD simulations of multi-passes nanoscratching of single crystalline copper. The experimental and theoretical methodologies of nanoscratching are mainly consistent with each other, as the nanoscratching tests are performed under the load-controlled mode using a triangular pyramidal probe. We elucidate the microscopic deformation mechanisms of single crystalline copper, and also reveal their correlations with the macroscopic machining results in terms of machining force and machined surface quality. We further examine the crystallographic orientation dependence of the nanoscratching.

## Methodologies

### 2.1 Experiments

**Fig 1:**
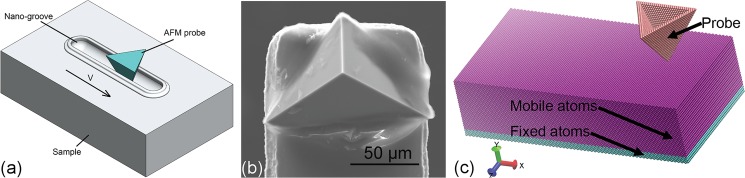
Load-controlled multi-passes nanoscratching. (a) schematic illustration of nanoscratching experiment configuration; (b) SEM image of triangular pyramid-shaped diamond probe; (c) MD model of nanoscratching.

The probe-based nanoscratching experiments are carried out in a commercial AFM-based nanomachining system (Dimension Icon, Bruker Company, Germany). Particularly, the equipped Nanoman module in the AFM system is employed for the multi-passes scratching. Fig 1(A) illustrates the configuration of multi-passes nanoscratching experiment. Each scratching pass can be divided into three sequential phases, as first penetration, following scratching and final retraction, respectively. In the first phase of penetration, a pre-determined normal load *F*_*N*_ is applied to the cantilever with a normal spring constant (*K*_N_) of 275 N/m, which results in a diamond probe (PDNISP, Bruker Company, Germany) penetrating into the sample surface under the contact mode of the AFM. The triangular pyramidal diamond probe shown in Fig 1(B) has a radius of approximately 110 nm evaluated by the blind reconstruction method [26]. In the second phase of scratching the applied normal load (*F*_*N*_) is maintained, and the probe driven by the piezoelectric ceramic transducer scratches 20 μm with a constant velocity of 3 μm/s along the direction highlighted by the red arrow shown in Fig 1(A), which leads to a straight groove of nanometer dimensions formed on the sample surface. To minimize the error of applied normal load between pre-determined and actual values, the scratching direction perpendicular to the cantilever is particularly chosen. In the third phase of retraction, the probe returns to its initial position prior to the first penetration with a constant velocity of 10 μm/s under the tapping mode of the AFM. In the present work, the number of scratching passes is fixed as 3 for each normal load. After completion of the multi-passes scratching, the machined sample surface is first ultrasonically washed in alcohol solution for 10 min to remove chips formed in the scratching process, and then subjected to imaging in the AFM under the tapping mode using a silicon nitride probe with a probe radius of 10 nm.

To reveal the crystallographic orientation dependence of material removal in the nanoscratching, three commercially-provided single crystalline copper samples with different surface orientations of (010), (110) and (111) are considered. The 2θ-diffraction angles obtained from the X-Ray diffraction for the (010)-, (110)- and (111)-oriented single crystalline copper are 50.4, 43.36 and 74.18^。^, respectively. All the three copper samples have the same dimension of 10 mm × 10 mm × 1 mm, and the measured surface roughness (*R*_a_) is less than 5 nm. For each sample, six normal loads (20.6, 28.9, 37.2, 45.4, 53.7 and 61.9 μN) are considered.

### 2.2 Simulations

In addition to the nanoscratching experiments, MD simulations of load-controlled multi-passes nanoscratching of single crystalline Cu(010), (110) and (111) surfaces are also performed by using the LAMMPS code with an integration timestep of 1 femtosecond [27]. Fig 1(C) presents the MD model of the nanoscratching, which consists of a copper sample and a triangular pyramidal diamond probe. Each sample has a dimension of 28 nm, 7 nm and 14 nm in X, Y and Z directions, respectively. Periodic boundary conditions are only applied in lateral X and Z directions, and the bottom of the sample along Y direction is fixed in space. The atomic interactions in copper sample are described by an EAM potential developed for Cu [28], and the interactions between carbon atoms and copper atoms are described by the Morse potential [29].

Prior to the nanoscratching, the as-created samples are first equilibrated at 30 K in the NVT (constant number of atoms, volume and temperature) ensemble for 50 ps. Then the relaxed sample is subjected to the nanoscratching in the NVT ensemble at 30 K. The relative low temperature of 30 K is used to eliminate thermal effect on the nanoscratching [30]. To be consistent with experimental configuration, the multi-passes nanoscratching simulation also consists of three phases, as initial penetration along negative Y direction, following scratching along negative X direction and final retraction to its initial position before penetration, respectively. Both the penetration and scratching are carried out in the load-controlled mode by applying constant normal load on the probe. In the first phase of penetration with a duration of 20 ps, a normal load of 56 nN is applied to the probe. In the following scratching phase the constant normal load is maintained, while the probe scratches 16 nm on the sample surface with constant velocity of 100 m/s. In the final phase of retraction the applied normal load is fully released, and the probe first moves upwards along positive Y direction for 4 nm, and then return to its initial position along positive X direction. To make the computational time reasonable, a relative high scratching velocity is used in the present MD simulations, given the intrinsic limitation of the integration timestep to be in the order of the magnitude of fs. The common neighbor analysis (CNA) is employed to distinguish different types of defects generated in the samples [31]. The Ovito is utilized to visualize MD data and generate MD snapshots [32].

## Results and Discussion

### 3.1 Mechanisms of multi-passes nanoscratching

**Fig 2:**
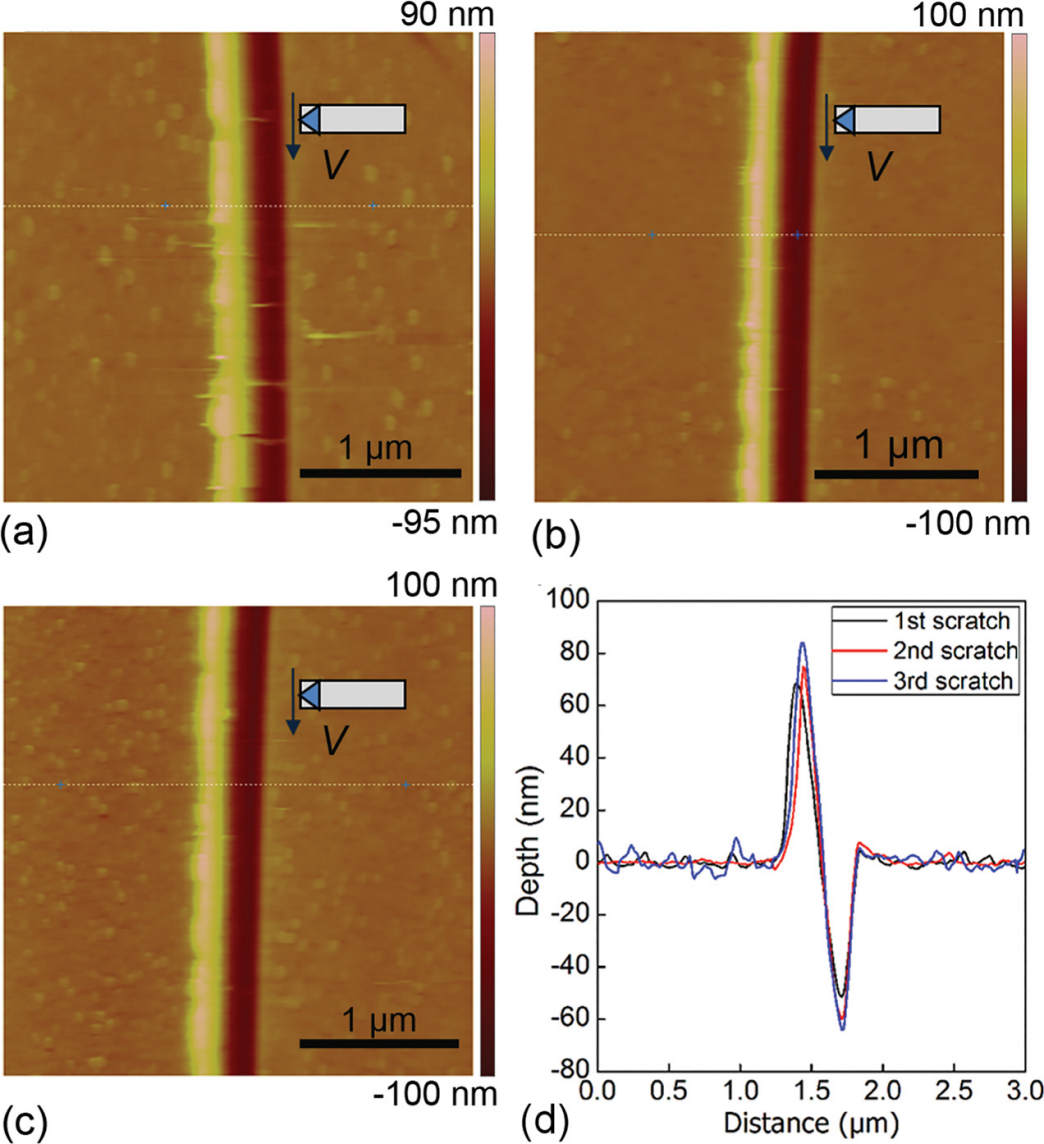
Machined surface morphologies of single crystal Cu(010) after multi-passes nanoscratching. AFM image of the machined surface after (a) 1st, (b) 2nd and (c) 3rd scratching passes. The scratching direction is highlighted by the arrow. (d) Profiles of multi-passes scratching-induced grooves.

Fig 2(A)–2(C) present AFM images of machined surface morphologies of single crystalline Cu(010) after the 1st, 2nd and 3rd scratching passes under a normal load of 37.2 μN, respectively. For each scratching pass, the scratching direction is the same as [100]. Fig 2(D) further quantitatively characterizes the profiles of multi-passes nanoscratching-induced grooves. It is found from Fig 2 that for each scratching pass, there is V-shaped groove formed on the sample surface, and the accumulation of surface pile-up mainly resides on the left side of the groove. Fig 2(D) demonstrates that for each scratching pass, either height or volume of the surface pile-up is larger than that of the groove, indicating that the material removal is primarily achieved through accumulation of surface pile-up, rather than formation of chips [33–34]. Fig 2(D) also suggests that the influence of scratching pass on the groove profile is not trivial. With the increase of scratching pass number, both the height of surface pile up and the depth of groove increase.

**Fig 3:**
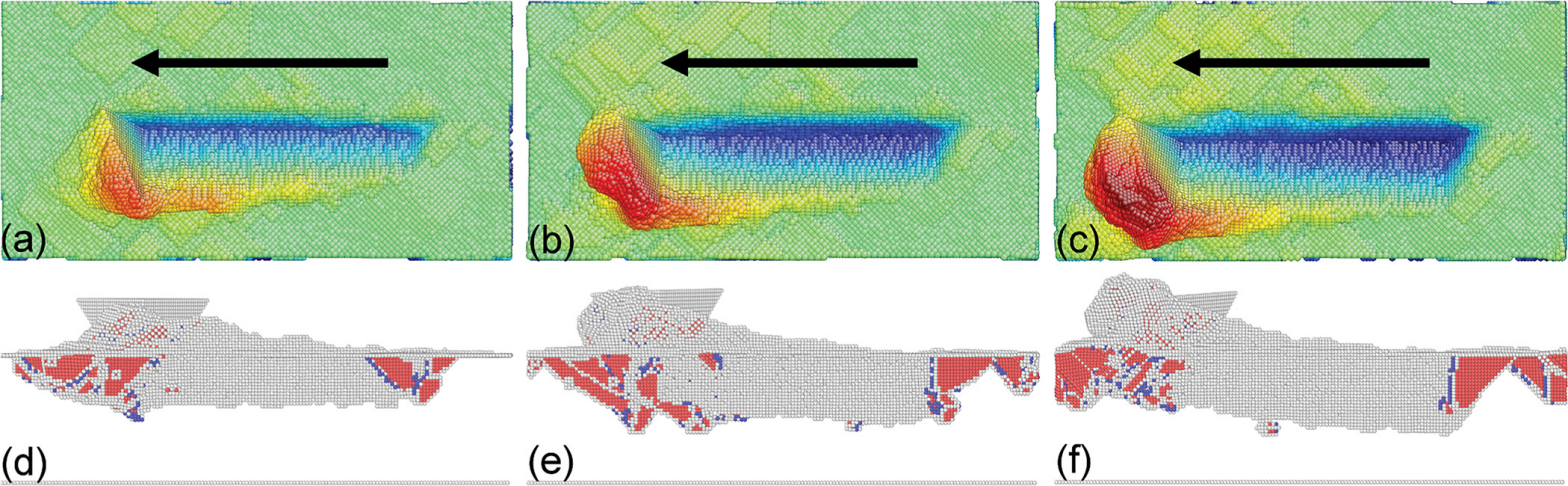
MD snapshots of load-controlled multi-passes nanoscratching of single crystalline Cu(010). Top and bottom rows show machined surface morphologies and instantaneous defect structures after (a) and (d) 1st, (b) and (e) 2nd, and (c) and (f) 3rd scratching pass. Atoms in the top and bottom rows are colored according to their atomic heights and CNA values, respectively.

As a supplement to the experiments, MD simulations of multi-passes nanoscratching of single crystalline Cu(010) along [100] direction is also performed. The top row in Fig 3 presents machined surface morphologies of the Cu sample after different scratching passes. The scratching direction is indicated by the arrow. It is seen from Fig 3(A)–3(C) that there is no chip formed and the surface pile-up mainly accumulates on the left side of the groove, which are consistent with the experimental observations. Particularly, it is also found that the displaced material in front of the probe adheres closely to one side of the triangular pyramid, indicating the material accumulation is strongly influenced by the probe geometry. The bottom row in Fig 3 presents instantaneous defect structures within the sample after different scratching passes. It is found from Fig 3(D)–3(F) that there are considerable dislocations generated in the material after each scratching pass. Furthermore, the dislocation density increases with scratching pass number.

**Fig 4:**
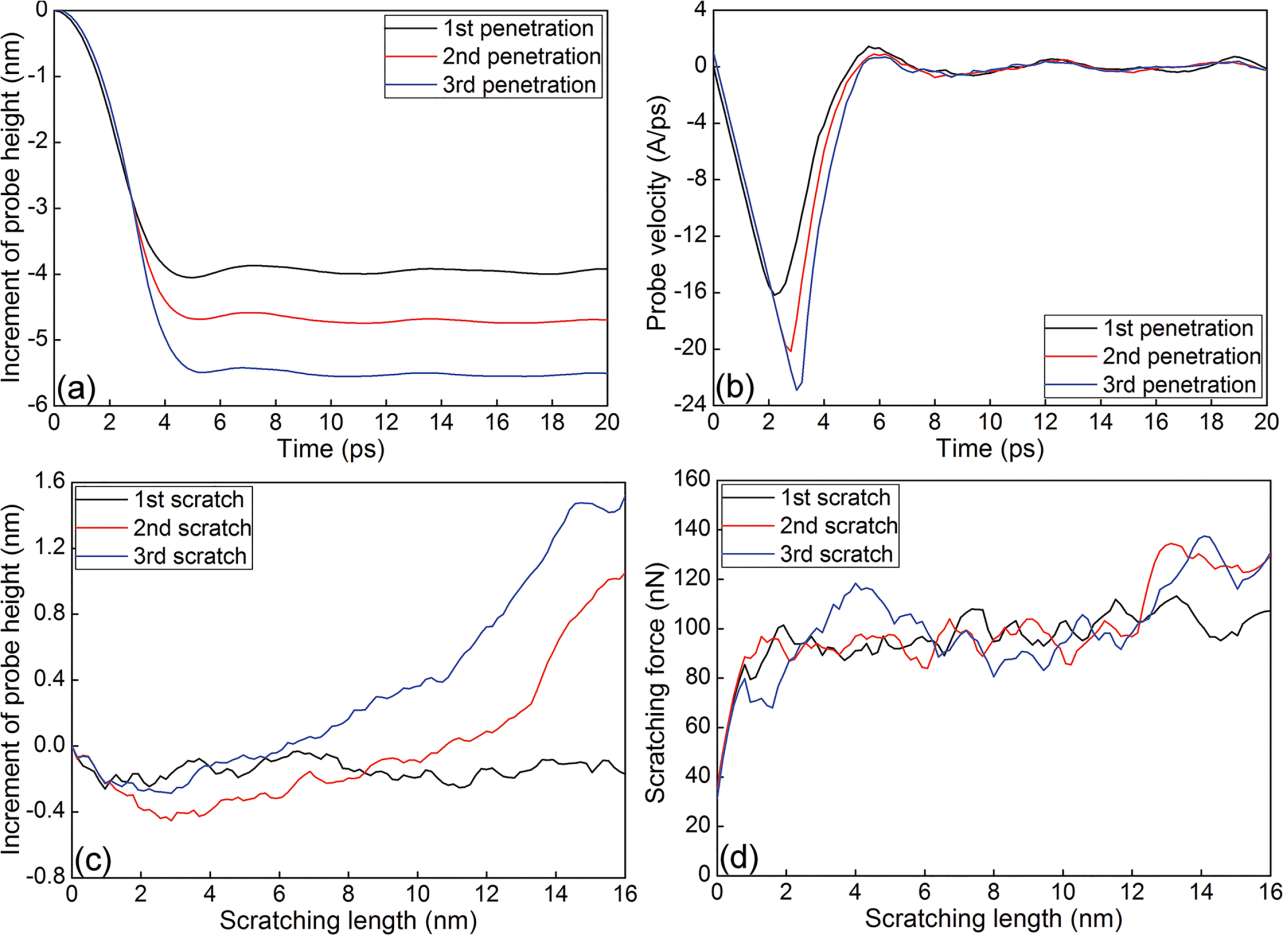
Mechanical response during the load-controlled multi-passes nanoscratching of single crystalline Cu(010). (a) Increment of probe height-time curves and (b) Probe velocity-time curves during the penetration phase. (c) Increment of probe height-scratching length curves and (d) Scratching force-scratching length curves during the scratching phase.

Fig 4(A) and 4(B) plot the variations of the increment of probe height and velocity with time during the penetration phases of multi-passes nanoscratching of single crystalline Cu(010) along [100] direction, respectively. In the penetration phase, the increment of probe height is calculated by subtracting the probe height before penetration using the current height. For the first scratching pass, the magnitude of probe velocity first increases linearly accompanied by the probe approaching to the sample surface until the initial contact occurs, at which the increase of probe velocity reverses. However, the probe height continues to decrease due to the probe penetrating into the sample surface. Fig 4(A) shows that after the probe height reaches its lowest value, the probe first bounces upwards slightly and then fluctuates around a constant value, accompanied with the fluctuation of probe velocity around 0, indicating that the penetration is stable. Fig 4(A) and 4(D) plot variations of the increment of probe height and scratching force with scratching length, respectively. In the scratching phase, the increment of probe height is calculated by subtracting the probe height before scratching using the current height. It is found that the probe height during the first scratching pass mainly keeps constant value and is lower than the value after the completion of penetration phase. Fig 4(D) shows that the scratching force first increases rapidly and then fluctuates around a constant value of 90 nN when scratching is stable. The strong fluctuation phenomena observed in Fig 4(D) can be attributed to dislocation nucleation events.

Fig 4 also demonstrates that the scratching pass number has significant influence on the mechanical response of nanoscratching. In the penetration phase, the constant value of probe height, the maximum value of probe velocity and the slope of velocity decreasing increase with scratching pass number. In the following scratching phase, the bouncing back phenomenon is more pronounced for larger scratching pass number. For either the second or the third scratching pass, Fig 4(C) shows that the probe height first decreases slightly in the initial scratching length of 3 nm, but increases significantly in the rest of scratching length. Furthermore, for each scratching pass number, the penetration depth is decreasing with increasing scratching length. It is seen from Fig 4(C) that after completion of the scratching phase, the increment of probe height is 1.0 and 1.5 nm for the second and third scratching pass number, respectively. Accordingly, Fig 4(D) shows that the constant value of scratching force is nearly independent on the scratching pass.

**Fig 5:**
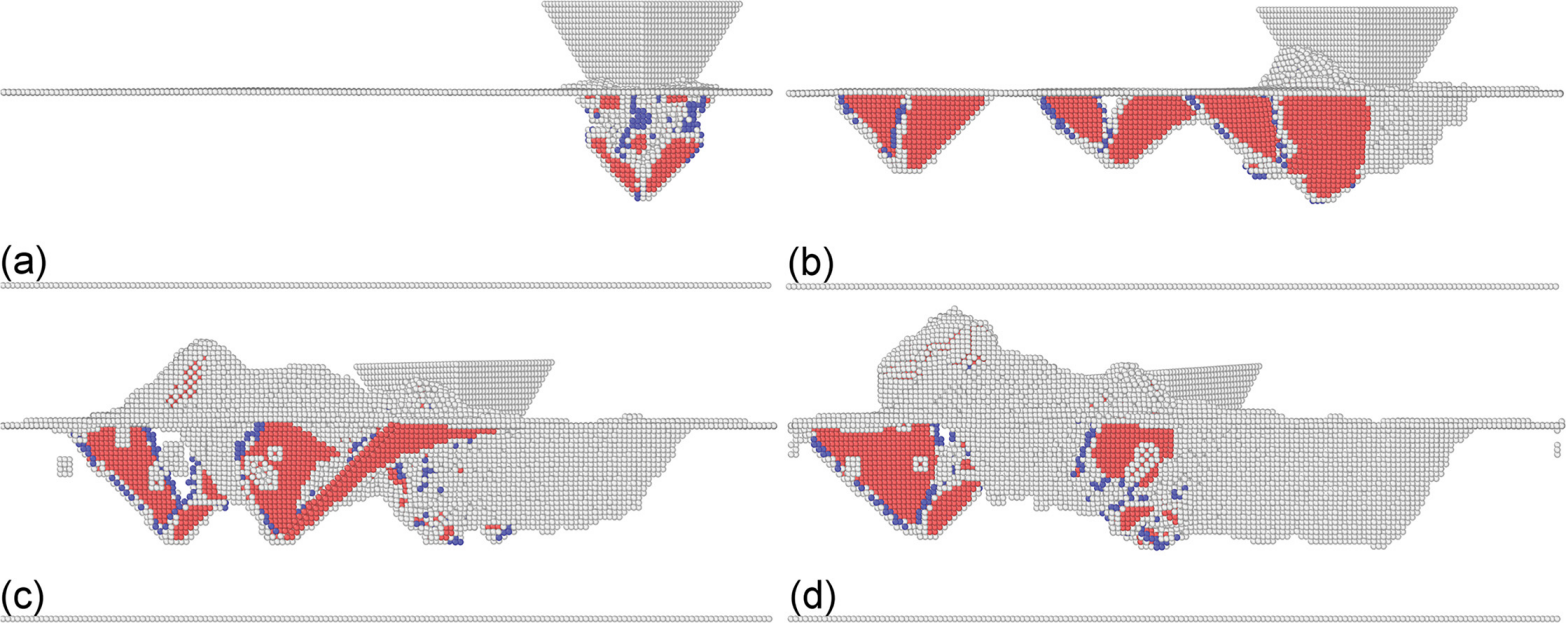
MD snapshots of defect evolution during load-controlled multi-passes nanoscratching of single crystalline Cu(010). Instantaneous defect structures after the (a) penetration and (b) scratching phase of the first scratching pass. Instantaneous defect structures after the scratching pass of the (c) 2nd and (d) 3rd scratching pass.

To reveal the underlying deformation mechanisms of single crystalline Cu(010), dynamic analysis of defect evolutions during the load-controlled multi-passes nanoscratching is carried out. Fig 5 presents representative MD snapshots of instantaneous defect structures during the nanoscratching process. For the single scratching pass, the deformation mechanisms of single crystalline Cu(010) can be summarized as follows: in the penetration phase, the defect-free material first undergoes first elastic deformation, followed by plastic deformation initiated by the nucleation of dislocations from the penetrated surface. In the plastic deformation the nucleated dislocations subsequently glide on {111} slip planes below the (010) free surface, and their reaction and cross-slip lead to the formation of sessile dislocation structures shown in Fig 5(A). However, there is no glissile dislocation structure such as prismatic dislocation loop formed, which is significantly different from previous MD simulations of the displacement-controlled penetration [30]. The discrepancy of dislocation structures between different penetration modes can be attributed to the oscillating displacement of the probe in the load-controlled mode, which releases accumulated plastic strain in the vicinity of the probe. In the following scratching phase, dislocation evolution is much more complex than that in the penetration phase because of more complex stress state of multi-axis. It is found from Fig 5(B) that while there are fresh dislocations nucleated from free surface in front of the probe due to the shear stress applied by the probe, dislocation annihilations occur at the free surface behind the probe because of the release of applied normal stress. Furthermore, there is still no glissile prismatic dislocation structures formed within the material in the scratching phase. Correspondingly, there are considerable displaced material accumulated on the sample surface, due to the glide of dislocations on {111} slip planes towards the surface and the annihilation of dislocation at the surface [30]. In the final retraction phase, the release of applied stress leads to considerable strain energy recovery, and the defect zone beneath the free surface shrinks slightly.

Be similar to the single scratching pass, the plastic deformation behavior of the Cu(010) under multi-passes nanoscratching is also dominated by dislocation mechanisms. However, Fig 5(C) and 5(D) demonstrate that the microscopic deformation behavior as well as the machining results is strongly dependent on the scratching pass number. In the penetration phase of multi-passes nanoscratching, the initiation of plastic deformation is governed by the motion of pre-existing dislocations beneath the scratched surface, instead of initial dislocation nucleation from the free surface. Since there are considerable sessile dislocations formed in the first scratching pass that act as barriers for dislocation motions, the strain hardening becomes more pronounced in the multi-passes nanoscratching. And the larger the scratching pass number, the more pronounced the strain hardening. Consequently, the probe height in the scratching phase is larger than the penetration phase, and increment of probe height increases with increasing scratching pass number, as shown in Fig 4(C).

**Fig 6:**
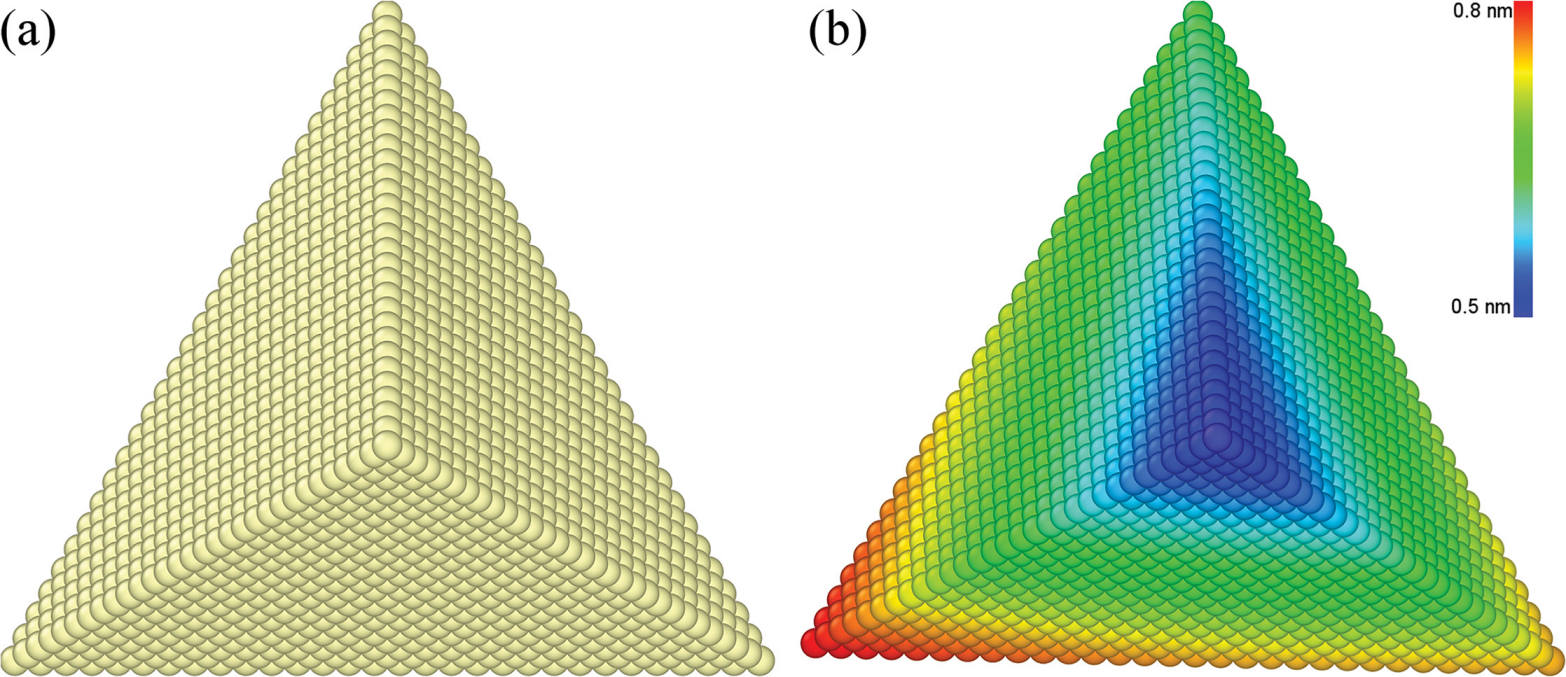
Inclination of the probe. Probe configuration (a) before nanoscratching and (b) after the 3rd scratching pass. Atoms in (b) are colored according to their displacement magnitudes.

It is also seen from Fig 3 that during the multi-passes nanoscratching the triangular pyramid-shaped probe is inclined, as compared to its original geometry. And the inclination angle increases with scratching pass number. To characterize the inclination of the probe, the initial configuration of the probe shown in Fig 6(A) is chosen as the reference to obtain the displacement magnitude of the probe after the nanoscratching. Fig 6(B) presents the atomic configuration of the probe colored by atomic displacement magnitudes after the third scratching pass, indicating that the inclination of the probe is composed of lateral displacement and radial rotation. And the atomic displacement magnitude of the probe increases with atomic height. The inclination of probe during the load-controlled multi-passes nanoscratching can be attributed to the heterogeneous deformation occurred within the material. It’s should be noted that in the experiments, the slight rotation and oscillation of the probe are eliminated after each scratching pass because of the large normal spring constant of the cantilever.

### 3.2 Effect of crystal orientation on the nanoscratching

**Fig 7:**
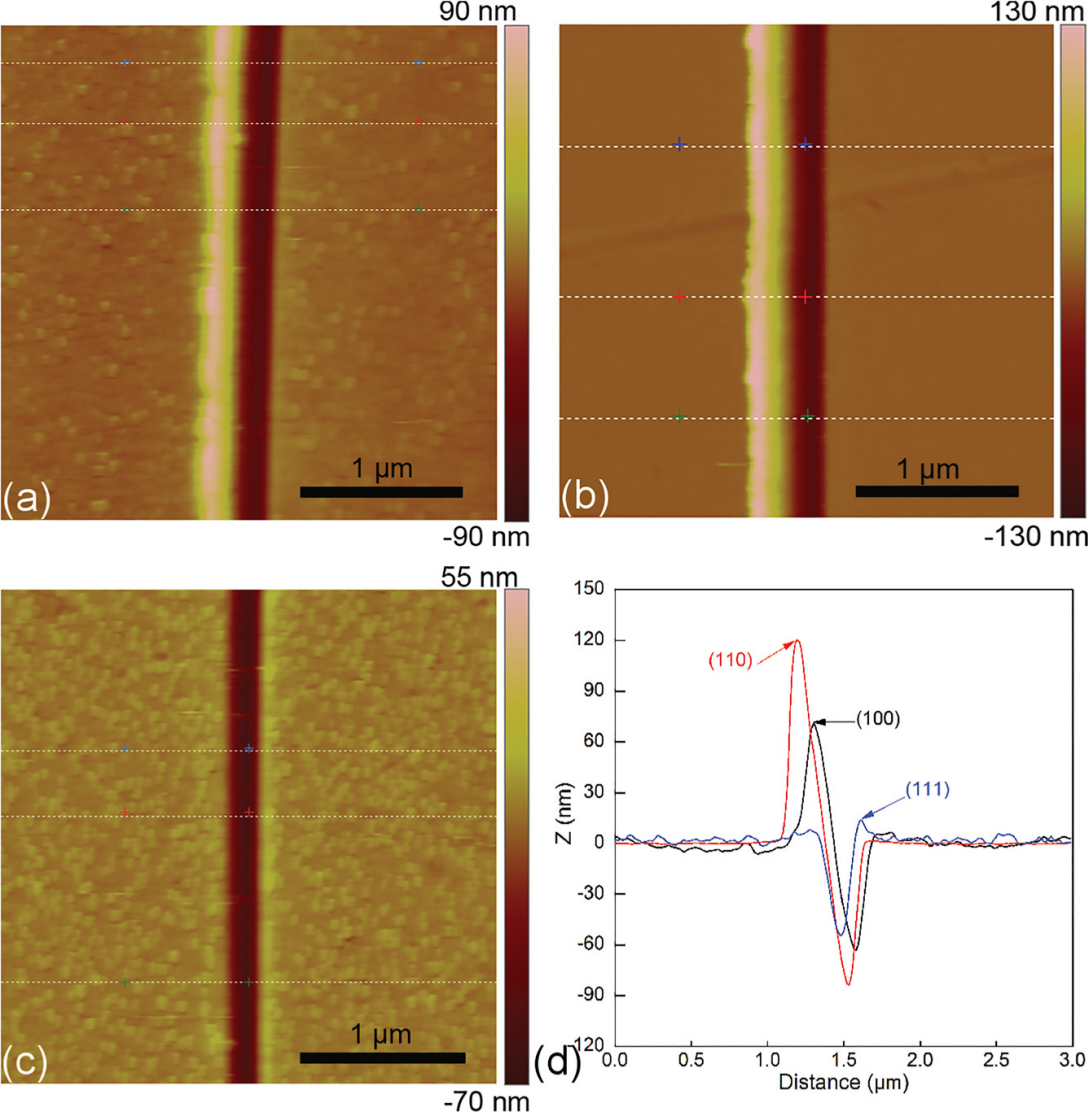
Machined surface morphologies of Cu samples after the 3rd scratching pass. AFM images of the machined surface of (a) (010), (b) (110) and (c) (111) orientations; (d) presents groove profiles of Cu samples with different orientations.

With the insights into the nanoscratching mechanisms of single crystalline Cu(010) obtained in the Section 3.1, the influence of crystallographic orientation on the nanoscratching is further examined by performing experiments and MD simulations of nanoscratching on Cu(110) along [110] direction and Cu(111) along [112] direction. The parameters used in the nanoscratching experiments are the same for each sample. Fig 7(A), 7(B) and 7(C) present AFM images characterizing the machined surface morphologies for Cu(010), Cu(110) and Cu(111) after the third scratching pass under a normal load of 37.2 μN, respectively. Furthermore, Fig 7(D) plots the profiles of scratching-induced grooves for the three Cu samples. For each sample, three random sampling positions shown in the corresponding AFM image are selected to obtain the cross-sectional profile of the groove, and the plotted groove height in Fig 7(D) is obtained by averaging the three heights accordingly. It is found from Fig 7 that for each crystallographic orientation, the multi-passes nanoscratching yields nearly uniform groove depths and surface pile-up heights at different positions along the groove. However, the machined surface quality is strongly dependent on the crystallographic orientation. Fig 7 shows that both groove depth and height of surface pile-up are the largest for the Cu(110), followed by the Cu(100), and the Cu(111). Furthermore, while the surface pile-up of Cu(010) and Cu(110) mainly resides on the left side of the groove, the surface pile up of Cu(111) is symmetrically distributed along the groove. In particular, there are continuous chips formed at the end of the groove for the Cu(111), which is consistent with previous experimental results [35].

**Fig 8:**
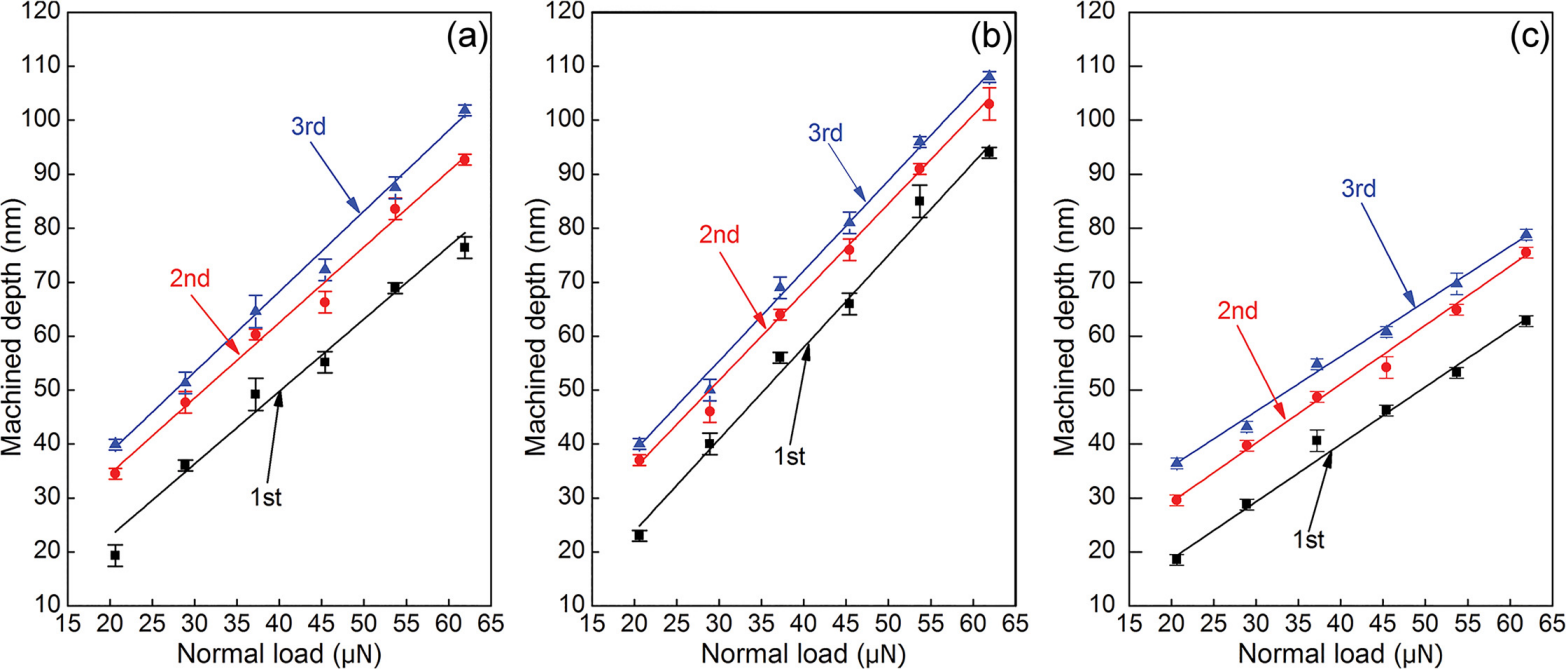
Normal load-dependent groove depth in multi-passes nanoscratching experiments of single crystalline Cu samples. Crystallographic orientations of (a) (010), (b) (110) and (c) (111).

Fig 8(A), 8(B) and 8(C) plot the depths of multi-passes scratching-induced grooves formed on the Cu(010), Cu(110) and Cu(111) under six different loads, respectively. In each scratching pass, measurements of the groove profile at three random positions are performed. The error bars shown in Fig 8 demonstrate good reproducibility of the scratching experimental data. It is found from Fig 8 that for each scratching pass, the groove depth is the largest for the Cu(110), followed by the Cu(100), and the Cu(111). Furthermore, Fig 8 demonstrates that the crystallographic orientation dependence of groove depth is not influenced by the applied normal load, as the linear fitting lines for different loads mainly parallel to each other.

**Fig 9:**
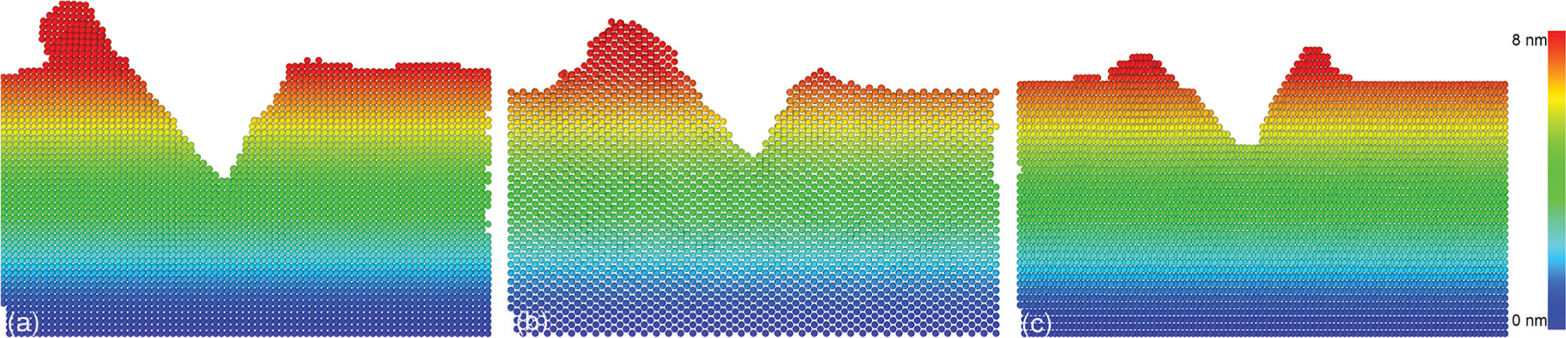
MD snapshots of cross-sectional view of groove profile after the 3rd scratching pass in multi-passes nanoscratching of single crystalline Cu samples. Crystallographic orientations of (a) (010), (b) (110) and (c) (111). Atoms are colored according to their atomic heights.

Be consistent with the experiments, MD simulations of multi-pass nanoscratching on Cu(110) along [110] direction and Cu(111) along [112] direction are also carried out. Fig 9(A), 9(B) and 9(C) present MD snapshots of cross-sectional view of groove profiles for the Cu(010), Cu(110) and Cu(111) after the 3rd scratching pass, respectively. It is found from Fig 9 that both the groove depth and the volume of surface pile up for the Cu(010) and Cu(110) are significantly larger than that for the Cu(111) [36]. Furthermore, while surface pile up is mainly accumulated on the left side of the groove for the Cu(010) and Cu(110), the material pile up symmetrically resides on the both sides of the groove for the Cu(111). Above theoretical observations are qualitatively consistent with the experimental results.

**Fig 10:**
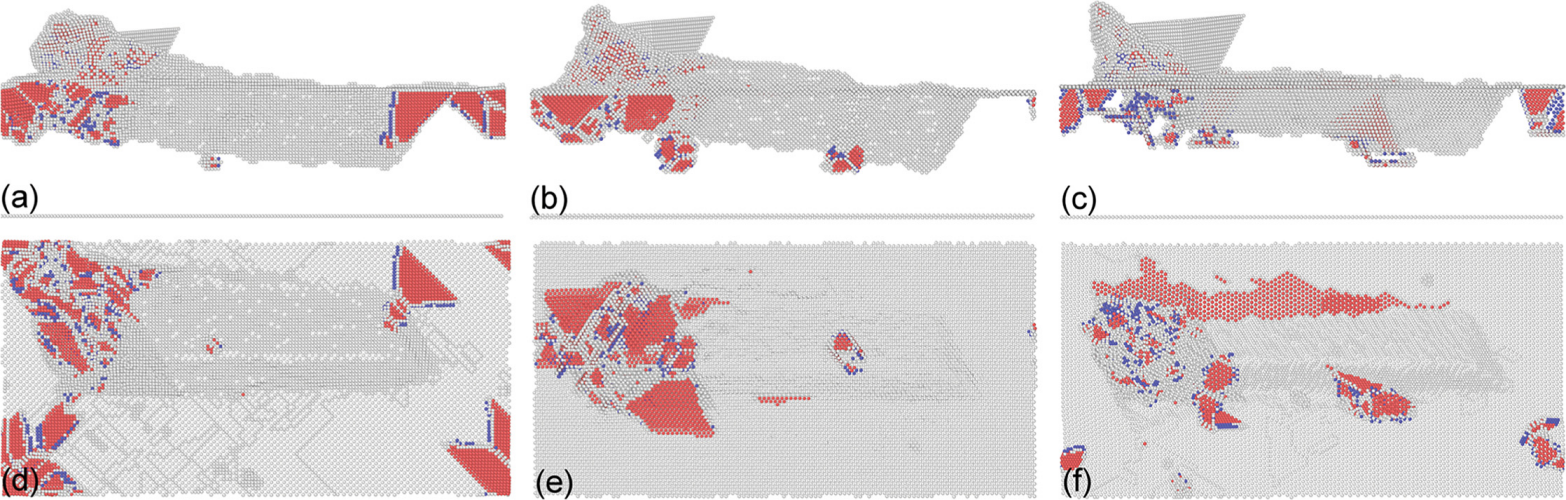
MD snapshots of instantaneous defect structures after multi-passes nanoscratching. The top row and bottom row show side and bottom views of instantaneous defect structures after the 3rd scratching pass. Crystallographic orientations of (a) and (d) is (010); (b) and (e) is (110); (c) and (f) is (111). Atoms are colored according to their CNA values, and perfect FCC atoms are not shown.

Fig 10 presents MD snapshots of dislocation structures within the three Cu samples after the 3rd scratching pass. It is seen from Fig 10 that for each Cu sample, there are considerable dislocations formed beneath the free surface that is in the vicinity of the probe, and the dislocation density in the rare of the probe is low due to dislocation annihilations at the free surface [30]. Fig 10 demonstrates that dislocation density is larger for the Cu(100) and Cu(110) than the Cu(111). Furthermore, the geometry between dislocation structures and free surface also varies with crystallographic orientation. For either the Cu(010) or Cu(110), dislocation structures are mainly inclined to the free surface, because of the activation of four {111} slip planes below the free surface. For the Cu(111) there are also dislocations inclined to the (111) free surface observed. However, there are considerable dislocations gliding parallel to the (111) free surface, because of the (111) slip plane parallel to the free surface.

**Fig 11:**
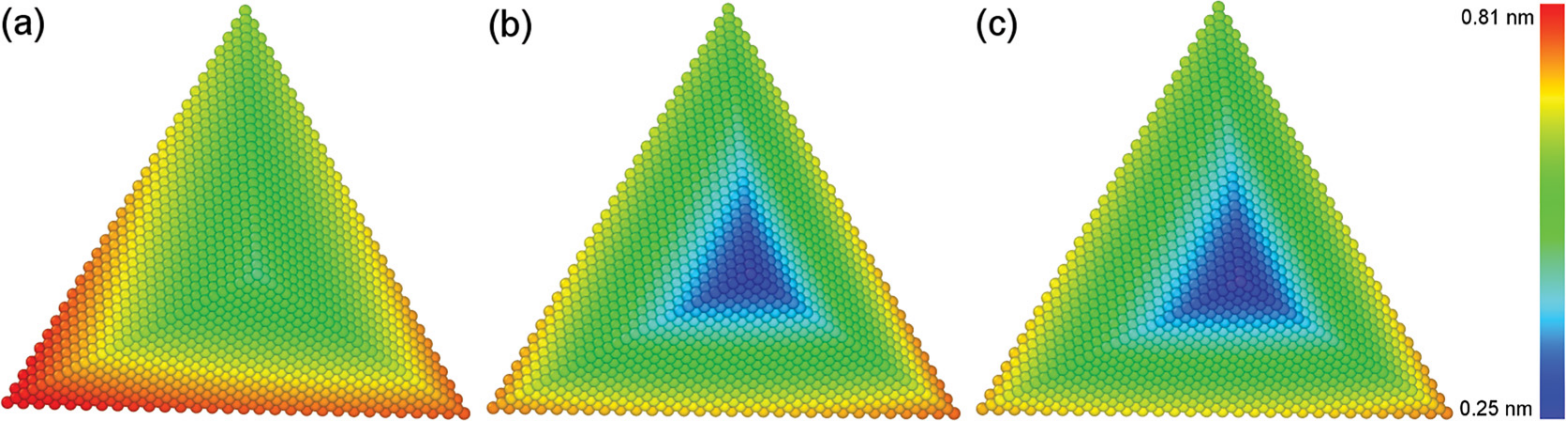
MD snapshots of inclined probe after multi-pass nanoscratching of single crystalline Cu samples. Crystallographic orientations of (a) (010), (b) (110) and (c) (111). Atoms are colored according to their atomic displacement magnitudes.

Fig 11(A), 11(B) and 11(C) present the inclination of the probe after multi-passes nanoscratching of the Cu(010), Cu(110) and Cu(111), respectively. It is found from Fig 11 that the displacement of the probe for the Cu(010) is significantly larger than the Cu(110) and Cu(111). For the Cu(100), the inclination of the probe is resulted by the lateral displacement of the bottom part and the radial rotation of upper part of the probe. For either the Cu(110) or Cu(111), however, the lateral displacement dominates the inclination of the probe.

Above experiments and MD simulations demonstrate a strong crystallographic orientation dependence of single crystalline Cu under the load-controlled multi-passes nanoscratching, which can be attributed to different geometries of activated slip planes with free surface and strain hardening abilities for different crystallographic orientations. MD simulations reveal that the plastic deformation of single crystalline Cu under the nanoscratching is exclusively governed by dislocation slip on {111} plane in <110> direction at ambient temperature. While there are 12 crystallographically equivalent {111}<110> slip systems in FCC copper, the yielding accompanied by the dislocation nucleation will only begin on one slip system when the applied resolved shear stress on the system first reaches a critical value. The activation of slip systems can be determined by calculating the Schmid factor *M* = cos *φ* * cos *λ*, where *φ* and *λ* are the angle between the applied load direction and the slip plane normal and the angle between the applied load direction and the slip plane direction. In particular, the maximum Schmid factor indicates the primary slip system that carries the local deformation process.

In the scratching phase of the nanoscratching, there are multi-axis stress components acting on the sample, the Schmid factor is divided into two components: (i) a scratching Schmid factor associated with frictional resistance along the negative X direction and (ii) a normal Schmid factor associated with normal pressure along the negative Y direction. Table 1 lists the calculated maximum Schmid factors for the three crystallographic orientations, indicating that the activation of primary slip system highly depends on the orientation of the crystal axes relative to the applied load direction. For the Cu(100), all the four {111} planes beneath the (010) free surface are primary slip planes. However, there are only two primary slip planes for either the Cu(110) or Cu(111). Specifically, the primary slip planes for the Cu(110) are (111) and (11–1), for the Cu(111) are (1–11) and (-111). Furthermore, the geometry of {111} slip planes cutting free surface varies with crystallographic orientation. Consequently, the morphology of scratched surface shows a strong dependence on crystallographic orientation, because the transportation of displaced material is primarily along the intersection between the primary slip plane and the scratched free surface [30,37–38]. We also note that the probe geometry also has significant influence on the surface pile-up pattern, as the displaced materials mainly flow through the side face of the probe parallel to the scratching direction.

**Table 1:** Maximum scratching and normal Schmid factors of the single crystal Cu with different crystallographic orientations.

Crystallography	(100)	(110)	(111)
Scratching	0.471/4	0.471/2	0.392/2
Normal	0.471/4	0.471/2	0.314/3

It is also seen from Table 1 that there are at least two primary slip planes activated for each crystallographic orientation under the scratching phase. The multiple slips on neighboring slip planes resulting in dislocation multiplication, which leads to dislocations blocking the motion of each other because of the decreased average distance between them. Furthermore, the cross-slip and reaction of dislocations lead to formation of the sessile dislocation structures shown in Fig 3, which act as barriers to dislocation motion. Consequently, significant strain hardening occurs in the material, which leads to the decrease of scratching depth with increasing scratching number, given the maintained constant normal load. Furthermore, since the strain hardening is associated with the increase of dislocation density with plastic deformation due to multiplication, there is also a strong crystallographic orientation dependence of the strain hardening ability because of different primary slip systems for different crystallographic orientations.

## Summary

In summary, we perform experiments and MD simulations of the load-controlled multi-passes nanoscratching of single crystalline copper using a triangle pyramidal probe, with an emphasis on the influence of crystallographic orientation on the nanoscratching. Our results show that dislocation slip is the dominant deformation mode operating in the plastic deformation of the Cu materials. However, there is no glissile dislocation structure formed thorough the nanoscratching process, because of the oscillation of the probe. While the plastic deformation of the material in the first scratching pass is initiated by the nucleation of fresh dislocation, the onset of plasticity in the multiple scratching passes is governed by the motion of pre-existing dislocations. The heterogeneous plastic deformation within the material leads to the inclination of the probe. It is found that both the surface pile-up height and groove depth have a strong dependence on the crystallographic orientation. While the surface pile up for either the Cu(010) or Cu(110) mainly resides on the left side of the groove, the surface pile up for the Cu(111) is symmetrically distributed along the groove. Moreover, the crystallographic orientation dependence of nanoscratching of single crystalline Cu is not influenced by the applied normal load. However, the inclination degree of the probe are larger for the Cu(010) than that for both the Cu(110) and Cu(111).

## References

[1] Suchowski H, O’Brien K, Wong ZJ, Salandrino A, Yin XB, et al (2013) Phase Mismatch-Free Nonlinear Propagation in Optical Zero-Index Materials. Science 342(6163):1223–1226. 10.1126/science.1244303 24311687

[2] Liu H, Yadian B, Liu Q, Gan CL, Huang Y (2013) A hybrid nanostructure array for gas sensing with ultralow field ionization voltage. Nanotechnology 24(17): 175301 10.1088/0957-4484/24/17/175301 23548746

[3] Nazirzadeh Y, von Oertzen F, Karrock T, Greve J, Gerken M (2013) Enhanced sensitivity of photonic crystal slab transducers by oblique-angle layer deposition. Opt Express 21(16):18661–18670. 10.1364/OE.21.018661 23938782

[4] Tak M, Gupta V, Tomar M (2014) Flower-like ZnO nanostructure based electrochemical DNA biosensor for bacterial meningitis detection. Biosens Bioelectron 59:200–207. 10.1016/j.bios.2014.03.036 24727606

[5] Fanzio P, Manneschi C, Angeli E, Mussi V, Firpo G, et al (2012) Modulating DNA Translocation by a Controlled Deformation of a PDMS Nanochannel Device. Sci Rep 2:791 10.1038/srep00791 23145315 PMC3494361

[6] Ciesielski PN, Wang W, Chen X, Vinzant TB, Tucker MP, et al (2014) Effect of mechanical disruption on the effectiveness of three reactors used for dilute acid pretreatment of corm stover Part 2: morphological and structural substrate analysis. Biotechnol Biofuels 7:47 10.1186/1754-6834-7-47 24690534 PMC4022059

[7] Wu SL, Wen L, Cheng GA, Zheng RT, Wu XL (2013) Surface Morphology-Dependent Photoelectrochemical Properties of One-Dimensional Si Nanostructure Arrays Prepared by Chemical Etching. ACS Appl Mater Interfaces 5(11):4769–4776. 10.1021/am400092w 23668230

[8] Podymova NB, Karabutov AA, Cherepetskaya EB (2014) Laser optoacoustic method for quantitative nondestructive evaluation of the subsurface damage depth in ground silicon wafers. Laser Phys 24(8):086003.

[9] Tseng AA (2011) Three-dimensional patterning of nanostructure using atomic force microscopy. J Vac Sci Technol B 29(4):040801.

[10] Yan Y, Geng Y, Hu Z, Zhao X, Yu B, et al (2014) Fabrication of nanochannels with ladder nanostructure at the bottom using AFM nanoscratching method. Nanoscale Res Lett 9(1):212 10.1186/1556-276X-9-212 24940171 PMC4039066

[11] Meyers MA, Mishra A, Benson DJ (2006) Mechanical properties of nanocrystalline materials. Prog Mater Sci 51(4):427–556.

[12] Singh A, Dao M, Lu L, Suresh S (2011) Deformation, structural changes and damage evolution in nanotwinned copper under repeated frictional contact sliding. Acta Mater 59(19):7311–7324.

[13] Zhang J, Wei Y, Sun T, Hartmaier A, Yan YD, et al (2012) Twin boundary spacing-dependent friction in nanotwinned copper. Phys. Rev. B 85:054109.

[14] Pitchuka SB, Lahiri D, Sundararajan G, Agarwal A (2014) Scratch-induced deformation behavior of cold-sprayed aluminum amorphous/nanocrystalline coatings at multi load scales. J Therm Spray Techn 23(3):502–513.

[15] Salehinia I, Bahr DF (2014) Crystal orientation effect on dislocation nucleation and multiplication in FCC single crystal under uniaxial loading. Int J Plasticity 52:133–146.

[16] Lin YC, Pen DJ, Chen JN (2014) Molecular dynamic simulation of stress evolution analysis in Cu nanowire under ultra-high strain-rate simple tension, Mol Phys 112(8):1115–1122.

[17] Tschopp MA, McDowell DL (2007) Tension-compression asymmetry in homogeneous dislocation nucleation in single crystal copper. Appl Phys Lett 90:121916.

[18] Wu B, Heidelberg A, Boland JJ, Sader JE, Sun X, et al (2006) Microstructure-Hardened Silver Nanowires. Nano Lett 6(3):468–472. 16522044 10.1021/nl052427f

[19] Zheng YG, Zhang HW, Chen Z, Wang L, Zhang ZQ, et al (2008) Formation of two conjoint fivefold deformation twins in copper nanowires with molecular dynamics simulation, Appl Phys Lett 92(4):041913.

[20] Zhang JJ, Yan YD, Liu X, Sun T, Liang YC (2014) Influence of coherent twin boundary on three-point bending of gold nanowires. J Phys D: Appl Phys 47(19):195301.

[21] Ju SP, Wang CT, Chien CH, Huang J (2007) The nanoindentation responses of nickel surfaces with different crystal orientations. Mol Simulat 33(11):905–917.

[22] Salehinia I, Bahr DF (2013) Mechanical behavior of FCC single crystals at finite temperatures in the presence of point defects. Mater Sci Eng: A 588:340–346.

[23] Komanduri R, Chandrasekaran N, Raff LM (2000) MD simulation of indentation and scratching of single crystal aluminum. Wear 240(1–2):113–143.

[24] Zhang JJ, Hartmaier A, Wei YJ, Yan YD, Sun T (2013) Mechanisms of anisotropic friction in nanotwinned Cu revealed by atomistic simulations. Modelling Simul Mater Sci Eng 21:065001

[25] Zhang P, Zhao H, Shi C, Zhang L, Huang H, et al (2013) Influence of double-tip scratch and single-tip scratch on nano-scratching process via moleculardynamics simulation. Appl Surf Sci 280:751–756.

[26] Dongmo LS, Villarrubia JS, Jones SN, Renegar TB, Postek MT, et al (2000) Experimental test of blind tip reconstruction for scanning probe microscopy. Ultramicroscopy 85:141–153.

[27] Plimpton S (1995) Fast Parallel Algorithms for Short-Range Molecular Dynamics. J. Comp. Phys. 117(1):1–19.

[28] Foiles SM, Baskes MI, Daw MS (1986) Embedded-atom-method functions for the fcc metals Cu, Ag, Ni, Pd, Pt, and their alloys. Phys Rev B 33: 7983. 10.1103/physrevb.33.7983 9938188

[29] Zhang JJ, Sun T, Yan YD, Liang Y,C Dong S (2009) Molecular dynamics study of groove fabrication process using AFM-based nanometric cutting technique. Appl Phys A 94(3):593–600.

[30] Zhang JJ, Sun T, Hartmaier A, Yan YD (2012) Atomistic simulation of the influence of nanomachining-induced deformation on subsequent nanoindentation. Comp Mater Sci 59:14–21.

[31] Honeycutt JD, Andersen HC (1987) Molecular dynamics study of melting and freezing of small Lennard-Jones clusters. J Phys Chem 91(19):4950–4963.

[32] Stukowski A (2010) Visualization and analysis of atomistic simulation data with OVITO-the Open Visualization Tool. Modelling Simul Mater Sci Eng 18:015012

[33] Tseng AA, Shirakashi J, Jou S, Huang JC, Chen TP (2010) Scratch properties of nickel thin films using atomic force microscopy. J Vac Sci Technol B 28:202–210.

[34] Tseng AA, Shirakashi JC, Nishimura S, Miyashita K, Notargiacomo A (2009) Scratching properties of nickel-iron thin film and silicon using atomic force microscopy. J Appl Phys 106:044314.

[35] Geng Y, Yan Y, Xing Y, Zhang Q, Zhao X, et al (2013) Effect of cantilecer deformation and tip-sample contact area on AFM nanoscratching. J Vac Sci Technol B 31(6):061802.

[36] Lu C, Gao Y, Deng GY, Michal G, Huynh NN, et al (2009) Atomic-scale anisotropy of nanoscratch behavior of single crystal iron. Wear 267(11):1961–1966.

[37] Wang Y, Raabe D, Kluber C, Roters F (2004) Orientation dependence of nanoindentation pile-up patterns and of nanoindentation microtextures in copper single crystals. Acta Mater. 52: 2229–2238.

[38] Gregory JG, Bogershausen H, Sander B, Raabe D (2010) Crystal orientation effects in scratch testing with a spherical indenter. J. Mater. Res. 25: 921–926.

